# Type 3 Adenylyl Cyclase and Somatostatin Receptor 3 Expression Persists in Aged Rat Neocortical and Hippocampal Neuronal Cilia

**DOI:** 10.3389/fnagi.2016.00127

**Published:** 2016-05-31

**Authors:** Sarah M. Guadiana, Alexander K. Parker, Gileno F. Filho, Ashton Sequeira, Susan Semple-Rowland, Gerry Shaw, Ronald J. Mandel, Thomas C. Foster, Ashok Kumar, Matthew R. Sarkisian

**Affiliations:** ^1^Department of Neuroscience, McKnight Brain Institute, University of FloridaGainesville, FL, USA; ^2^EnCor Biotechnology Inc.Gainesville, FL, USA

**Keywords:** aging, cerebral cortex, cilium, G protein-coupled receptor

## Abstract

The primary cilia of forebrain neurons assemble around birth and become enriched with neuromodulatory receptors. Our understanding of the permanence of these structures and their associated signaling pathways in the aging brain is poor, but they are worthy of investigation because disruptions in neuronal cilia signaling have been implicated in changes in learning and memory, depression-like symptoms, and sleep anomalies. Here, we asked whether neurons in aged forebrain retain primary cilia and whether the staining characteristics of aged cilia for type 3 adenylyl cyclase (ACIII), somatostatin receptor 3 (SSTR3), and pericentrin resemble those of cilia in younger forebrain. To test this, we analyzed immunostained sections of forebrain tissues taken from young and aged male Fischer 344 (F344) and F344 × Brown Norway (F344 × BN) rats. Analyses of ACIII and SSTR3 in young and aged cortices of both strains of rats revealed that the staining patterns in the neocortex and hippocampus were comparable. Virtually every NeuN positive cell examined possessed an ACIII positive cilium. The lengths of ACIII positive cilia in neocortex were similar between young and aged for both strains, whereas in F344 × BN hippocampus, the cilia lengths increased with age in CA1 and CA3, but not in dentate gyrus (DG). Additionally, the percentages of ACIII positive cilia that were also SSTR3 positive did not differ between young and aged tissues in either strain. We also found that pericentrin, a protein that localizes to the basal bodies of neuronal cilia and functions in primary cilia assembly, persisted in aged cortical neurons of both rat strains. Collectively, our data show that neurons in aged rat forebrain possess primary cilia and that these cilia, like those present in younger brain, continue to localize ACIII, SSTR3, and pericentrin. Further studies will be required to determine if the function and signaling pathways regulated by cilia are similar in aged compared to young brain.

## Introduction

Primary cilia are non-motile, microtubule-based organelles that extend from nearly every cell type in the body including neurons. They are believed to sense and respond to a variety of chemical changes in the local extracellular environment like small “cellular antennae” (for review see: Louvi and Grove, 2011; Guemez-Gamboa et al., 2014; Sarkisian and Guadiana, 2015). Throughout the mammalian cerebral cortex, most neurons are believed to possess a single primary cilium (Mandl and Megele, 1989; Fuchs and Schwark, 2004; Bishop et al., 2007; Anastas et al., 2011; Arellano et al., 2012; Guadiana et al., 2013), with the exception of gonadotropin-releasing hormone (GnRH) neurons which can possess multiple cilia (Jennes et al., 1985; Koemeter-Cox et al., 2014). In mouse cortex, the formation of neuronal cilia begins perinatally and continues for several months (Arellano et al., 2012). Neuronal cilia contain several signaling molecules, including adenylyl cyclase subtype III (ACIII; Berbari et al., 2007; Bishop et al., 2007; Arellano et al., 2012; Guadiana et al., 2013), the p75 nerve growth factor receptor (p75^NTR^; Chakravarthy et al., 2010, 2012a), as well as several G protein-coupled receptors (GPCRs) including melanin concentrating hormone receptor subtype 1 (MCHR1; Berbari et al., 2008a,b; Sun et al., 2012), serotonin receptor 6 (5HT_6_; Hamon et al., 1999; Brailov et al., 2000), somatostatin receptor subtype 3 (SSTR3; Händel et al., 1999; Berbari et al., 2008a; Domire and Mykytyn, 2009; Einstein et al., 2010), dopamine receptor 1 (D1; Marley and von Zastrow, 2010; Domire et al., 2011), kisspeptin receptor 1 (Koemeter-Cox et al., 2014) and neuropeptide Y receptors 2 and 5 (Loktev and Jackson, 2013).

There is growing evidence that suggests disruption of neuronal primary cilia structure and signaling capacity can induce defects in neuronal connectivity and reduce intellectual function (Einstein et al., 2010; Amador-Arjona et al., 2011; Wang et al., 2011; Kumamoto et al., 2012; Guadiana et al., 2013; Guo et al., 2015; Chen et al., 2016). Specifically, we have found that disruption of ciliogenesis in neocortical pyramidal neurons induces defects in dendrite growth and arborization that could be reversed by overexpression of ACIII in these neurons (Guadiana et al., 2013). Similar results have been obtained in recent studies of adult born dentate granule neurons. In these studies, ablation of ACIII+ cilia by expressing a dominant negative form of Kif3a in these granule cells disrupted their ability to develop dendrites and integrate into the adult brain (Kumamoto et al., 2012). Evidence that primary cilia dysfunction or loss alters neuronal and brain function comes from studies in which knockout of either ACIII or SSTR3 disrupts synaptic plasticity, behavior, and novel object recognition memory in mice (Einstein et al., 2010; Wang et al., 2011; Chen et al., 2016). Additionally, many ciliopathy patients present with cognitive impairments and other symptoms associated with CNS dysfunction (Valente et al., 2014; Guo et al., 2015).

Given the evidence linking primary cilia and brain function, and the complex mechanisms required to build and maintain a cilium (for reviews see: Ishikawa and Marshall, 2011; Keeling et al., 2016), in this study we explored whether neurons in the forebrains of aged rats are ciliated and whether these cilia retain ACIII and SSTR3 immunostaining profiles that resemble those observed in the forebrains of younger rats. We compared cilia in young and aged brains of male Fischer 344 (F344) and F344 × Brown Norway (F344 × BN) rats, commonly used rodent models in studies of brain aging.

## Materials and Methods

### Animals

Brain tissues analyzed in this study were derived from two strains of juvenile (2 mos, *n* = 4) and aged (aged 34 mos, *n* = 4) F344 × BN male rats and young adult (Kumar and Foster, 2013; 6 mos, *n* = 4) and aged (22–24 mos, *n* = 6) F344 male rats. Procedures involving animal subjects were reviewed and approved by the University of Florida Institutional Animal Care and Use Committee and were in accordance with guidelines established by the U.S. Public Health Service Policy on Humane Care and Use of Laboratory Animals.

### Immunohistochemistry

F344 × BN rats were intracardially perfused with saline followed by 4% paraformaldehyde in 0.1 M phosphate buffer (4% PFA), while F344 frontal cortices were dissected free from fresh tissue and were fixed by immersion in 4% PFA. Tissues were cryoprotected in sucrose, frozen over liquid N_2_, and sectioned coronally at 50 μm. Immunohistochemical analyses were performed using the following primary antibodies and a previously published protocol (Guadiana et al., 2013): rabbit anti-ACIII (1:10,000; Encor Biotechnology, #RPCA-ACIII), mouse anti-NeuN (1:2000; Chemicon, #MAB377), rabbit anti-Pericentrin (1:500; Covance, #PRB-432C), and goat anti-SSTR3 (1:200; Santa Cruz, #sc-11617). Appropriate species-specific, fluorophore-conjugated secondary antibodies were used to visualize binding of the primary antibodies (1:400; Jackson ImmunoResearch). To reduce lipofuscin autofluorescence, sections were treated with 0.3% Sudan Black in 70% ethanol for 10 min as previously described (Jackson et al., 2009). Stained sections were coverslipped with Prolong Antifade Gold mounting media containing DAPI (Life Technologies). Images of stained sections were captured on a Zeiss AxioObserver D1 epifluorescent microscope or an Olympus IX81-DSU spinning disc confocal microscope and are displayed as collapsed z-stacks that were collected in 1 μm steps.

### Cilia Analyses

The percentage of ciliated neurons was determined by placing the total number of ACIII+ cilia over the number of NeuN+ cells from each image. The percentage of SSTR3+ cilia was determined by placing the number of SSTR3+ cilia over the number of ACIII+ cilia. Only neurons with cell bodies and cilia within the field/z-stack were included in the analysis. To measure the lengths of the cilia, images were opened using Image J64^1^ and analyzed by tracing the ACIII immunostaining signal within the cilia. The lengths of cilia were measured in randomly selected images taken of neocortex (primary somatosensory or motor as indicated) and hippocampal subfields (dentate gyrus (DG), CA3, CA1) of each brain. Between 46 and 130 cilia were examined from six to eight fields per rat/region/age group in the F344 (*n* = 4 rats/group) and the F344 × BN (*n* = 4 rats/group) strains.

### Statistical Analysis

For each cortical area per animal, we calculated a mean cilia length and mean percentages of ciliated cells and co-labeled cilia. The averaged mean data from each brain region for each animal was statistically compared using a Student’s *t*-test with the level for significance set at *p* < 0.05.

## Results

### Expression of ACIII, A Marker of Cortical Neuronal Cilia, Persists in Aged Rats

**Figure 1:**
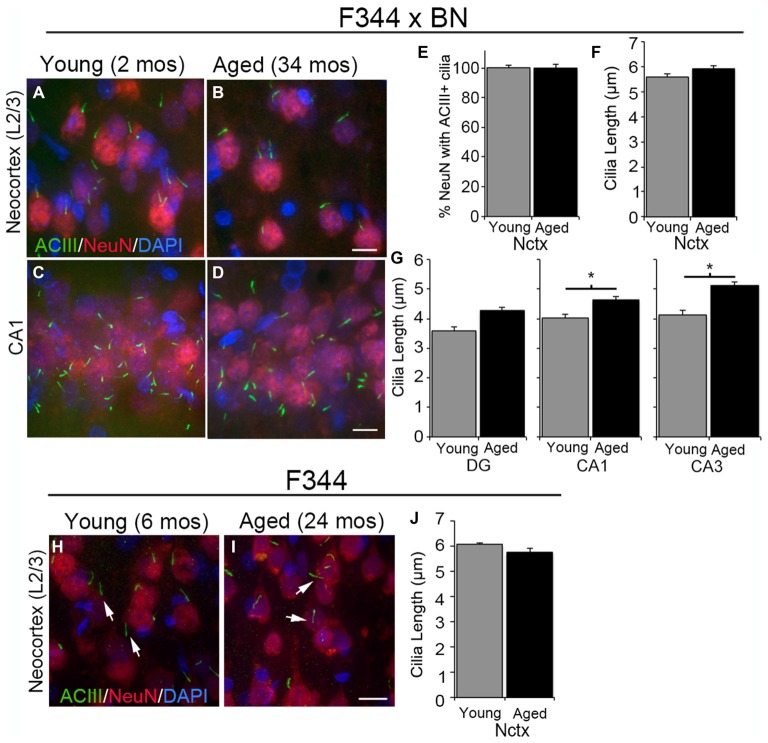
**Type 3 adenylyl cyclase (ACIII)-positive cilia in young and aged rat forebrain. (A–D)** Confocal images of 2 months (mos) (left panels) and 34 mos (right panels) Fischer F344 (F344) × Brown Norway (BN) rat layer 2/3 of neocortex (Nctx) **(A,B)** and CA1 region of hippocampus **(C,D)** immunostained for ACIII (green) and NeuN (red). Nuclei were stained with DAPI. **(E)** The percentage of NeuN positive cells also possessing ACIII positive cilia. **(F,G)** The lengths of ACIII positive cilia in the Nctx **(F)** and hippocampal subregions **(G)** of juvenile (*N* = 4) and aged (*N* = 4) rat brains. Data was statistically analyzed using Student’s *t*-test. *p < 0.05. **(H,I)** Confocal images of 6 and 24 mos F344 rat layer 2/3 of frontal neocortex immunostained for ACIII (green) and NeuN (red). Nuclei were stained with DAPI. **(J)** The lengths of ACIII positive cilia in 6 and 24 mos F344 Nctx. Scale bars in **B,D,I** = 10 μm.

We and others have shown that the primary cilia of neurons in the cortices of mice from birth to 2 years of age are enriched with ACIII (Bishop et al., 2007; Arellano et al., 2012; Chakravarthy et al., 2012a). In addition, we have shown that NeuN+ neurons in the neocortex of P14 Wistar rats possess one ACIII+ cilium (Anastas et al., 2011). To determine whether the neurons in the neocortices and hippocampi of aged rats possess ACIII+ cilia, we stained tissue sections of F344 × BN and F344 rats with antibodies recognizing ACIII and NeuN (Figures 1A–D,H,I). We found that nearly all of the neocortical NeuN+ cells that we examined possessed an ACIII+ cilium. No significant differences were observed in the numbers of neurons possessing ACIII+ cilia (percent ciliated) or the lengths of these cilia present in the neocortices of 2 month (mos) and 34 mos F344 × BN rats (Figures 1E,F). ACIII+ cilia were also readily detectable throughout the layers of the hippocampi of young and aged F344 × BN rats (Figures 1C,D). The ACIII+ cilia in CA1 and CA3 regions of hippocampus were significantly longer in aged compared to young brains (Figure 1G); this difference was not noted in comparison of the cilia within the DG. We also examined neurons in the frontal neocortices of 22–24 month old F344 rats to determine if their cilia were ACIII+. ACIII+ cilia were identified in the neocortices of these older F344 rats and the lengths of the cilia were not statistically different from those present in the neocortices of young F344 rats (Figures 1H–J). Taken together, our analyses of the neocortices and hippocampi of F344 × BN and F344 rats demonstrate that neurons in the rat forebrain maintain a primary cilium enriched with ACIII into late adulthood.

### Expression of SSTR3 in Cilia of the Young and Aged Forebrain

**Figure 2:**
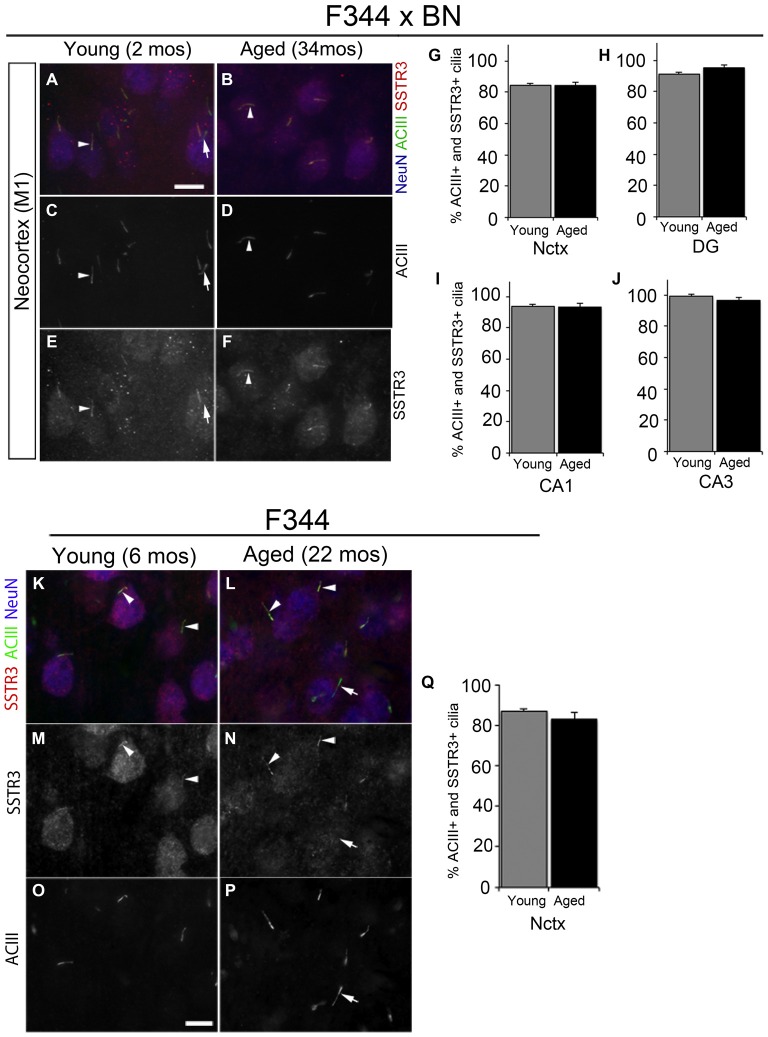
**Somatostatin receptor 3 (SSTR3)-positive cilia in young and aged rat forebrain. (A–F)** Epifluorescent images of 2 and 34 mos F344 × BN layer 2/3 primary motor neocortex (M1) co-immunolabeled for ACIII (green), SSTR3 (red) and NeuN (blue). Arrowheads indicate cilia positive for both SSTR3 and ACIII. **(G–J)** Percent of NeuN+ neurons that possess SSTR3+ cilia in M1 **(G)** and hippocampal DG **(H)**, CA1 **(I)** and CA3 **(J)** of 2 and 34 mos F344 × BN rats. **(K–P)** Images of 6 (left panels) and 24 mos (right panels) F344 rat layer 2/3 frontal neocortex immunostained for SSTR3 (red), ACIII (green) and NeuN (red). The arrowheads indicate cilia positive for both SSTR3 and ACIII, while the arrows **(A,C,E,L,N,P)** show examples of an ACIII positive cilium that is SSTR3 negative. **(Q)** Percent of NeuN positive neurons that possess SSTR3 positive cilia in frontal neocortex. Scale bars **(A,O)** = 10 μm.

The majority (~80–90%) of ACIII+ cortical neuronal cilia in the adult mouse brain also stain for SSTR3 (Bishop et al., 2007; Berbari et al., 2008b; Arellano et al., 2012; Guadiana et al., 2013). SSTR3+ cilia have also been reported to be present in the hippocampi of young adult (5 month old) rats (Stanić et al., 2009) and in 2 year old C57/BL6 mice (Chakravarthy et al., 2012a). To determine whether neuronal cilia in the cortices and hippocampi of aged rats are SSTR3+, we co-immunostained brain sections for SSTR3 and ACIII to label cilia, and NeuN to label neuronal cell bodies. First we compared the neuronal cilia present in the primary motor (M1) cortices, and the subfields of the hippocampi in the brains of young juvenile (2 mos) and aged (34 mos) F344 × BN rats (Figures 2A–F). We found that the majority of the ACIII+ cilia in the motor cortices of young and aged rats were also SSTR3+ (Figures 2G–I). A small number of ACIII+ cilia in both young adult and aged brain did not co-stain for SSTR3, a result consistent with our and others’ previous analyses (Green et al., 2012, 2016; Guadiana et al., 2013). We did not find significant differences between the percentages of SSTR3+ cilia in young and aged M1 cortices (Figure 2G). Similarly, we found that the majority of neuronal ACIII+ cilia in the CA1, DG, and CA3 subfields of the hippocampi of aging and young F344 × BN rats were also SSTR3+ (Figures 2H–J). We also examined the frontal neocortices of young adult (6 mos) and aged (22–24 mos) F344 rats and found that the majority of neuronal cilia co-labeled for ACIII and SSTR3 and that the percentages of neurons with ACIII+ and SSTR3+ cilia were not significantly different between young and aged brains (Figures 2K–Q). These results suggest that aging does not dramatically alter the expression or transport of SSTR3 into the primary cilia of neurons in rat neocortex and hippocampus.

### Pericentrin Localizes to the Base of Neuronal Cilia in Young and Aged Forebrain

**Figure 3:**
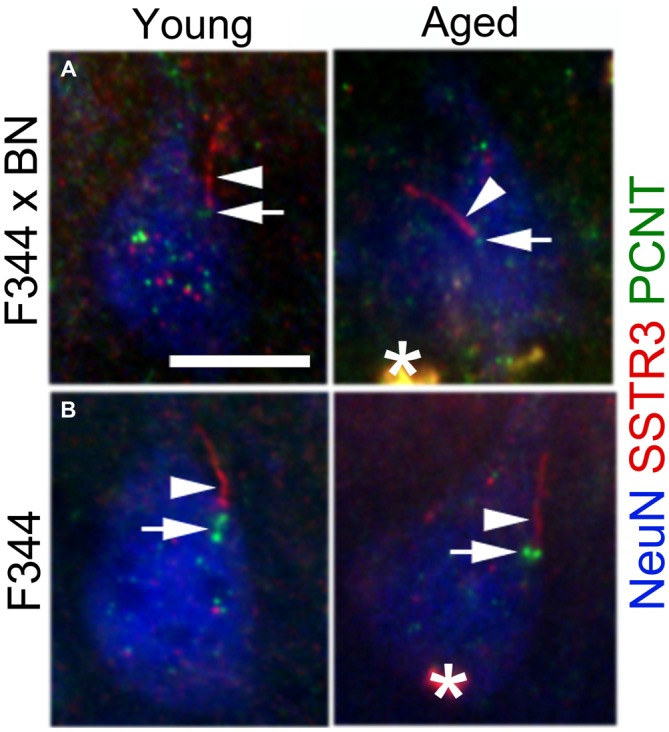
**Pericentrin localizes to the base of neuronal SSTR3+ cilia in young and aged rat cortex.** Sections of F344 × BN **(A)** and F344 **(B)** rat frontal cortex were immunostained for pericentrin (PCNT, green), SSTR3 (red), and NeuN (blue). Confocal images show PCNT at the base (arrows) of SSTR3+ cilia (arrowheads) in layer 2/3 neurons of both young (left panels) and aged (right panels) tissues. *lipofuscin. Scale bar = 10 μm.

The detection of SSTR3 along the lengths of aged cilia suggests that proteins associated with GPCR trafficking and intraflagellar transport (IFT) are still intact in the aged cortex. Pericentrin is a protein that localizes to the basal bodies of neuronal cilia (Arellano et al., 2012), and is reported to form a complex with IFT proteins that promote primary cilia assembly (Jurczyk et al., 2004). To determine if pericentrin expression persists in the aged cortex, we immunostained sections of young and aged F344 × BN and F344 frontal cortices for pericentrin, SSTR3 and NeuN. In both strains of rats, we detected pericentrin at the base of SSTR3+ cilia in neurons of both young and aged rats (Figure 3). This observation suggests that proteins associated with the ciliary IFT process are still trafficked to the cilia base in aged cortex.

## Discussion

Our results show that cortical neurons in the forebrains of aging F344 and F344 × BN rats retain primary cilia enriched with ACIII and SSTR3, signaling molecules that are present in the primary cilia of neurons in neonatal rat brain. Our findings are consistent with and extend those of several other groups who have characterized primary cilia in the brains of other aged rodents such as mice and hamsters (Suarez et al., 1985; Chakravarthy et al., 2010, 2012a,b), and in the brains of young adult (5 mos) Sprague-Dawley rats (Stanić et al., 2009). Recent reports indicate that altered cortical or hypothalamic neuronal cilia structure or loss of ciliary ACIII and SSTR3 can disrupt the neuronal development, cytoarchitecture, and function, and lead to altered behavior, learning and memory, and sleep (Einstein et al., 2010; Kumamoto et al., 2012; Guadiana et al., 2013; Han et al., 2014; Guo et al., 2015; Chen et al., 2016). In view of these reports, our results suggest that the aged cortex actively maintains its neuronal cilia, and that persistent compartmentalization of ACIII and SSTR3 in the cilium may be important for functional outcomes.

We and others have shown that ACIII is trafficked to and sequestered in the primary cilia of mouse neurons in mice ranging from birth to 1 year of age (Bishop et al., 2007; Arellano et al., 2012; McIntyre et al., 2015). In this study we have extended these observations to include the primary cilia of neurons in the brains of very old rats. ACIII’s functional role(s) in the primary cilia of cortical neurons is still unclear; however, recent studies suggest that it may be involved in regulating formation of the dendritic arbors of neurons in developing and adult forebrain (Kumamoto et al., 2012; Guadiana et al., 2013; Luo et al., 2015; Chen et al., 2016). The absence of ACIII has been linked to learning and memory deficits (Wang et al., 2011) and altered sleep patterns and pro-depression-like phenotypes (Chen et al., 2016). These observations strongly suggest that ciliary ACIII supports normal cognition and behavior. Additional studies will be needed to determine whether or not specific age-related cognitive impairments are associated with changes in the ACIII signaling pathway.

It is noteworthy that in aged F344 × BN cortex, we found that ACIII+ cilia were elongated in some hippocampal regions but not neocortex. The reason for this regional growth difference is unclear and could be attributable to multiple factors. One possibility is suggested by the results of a recent study that showed that loss of dopaminergic projections to striatal neurons can induce elongation of the primary cilia of these neurons (Miyoshi et al., 2014). Several studies of the hippocampus have shown that reductions in dopaminergic system input to the hippocampus occur with age (Gasbarri et al., 1996; Hemby et al., 2003; Wilson et al., 2006; Hernández et al., 2014). Further study will be required to determine if age-related changes in dopamine signals or dopaminergic input to the hippocampus underlie the elongated cilia phenotype that we observed in this rat strain. It is also possible that changes in cilia length in aged hippocampus could reflect age-related changes in the complex mechanisms governing primary cilia length control (for review see, Keeling et al., 2016). For example, loss of intestinal cell kinase, a negative regulator of cilia length, was reported to increase the rate of anterograde but not retrograde IFT, leading to increased cilia length in IMCD-3 cells (Broekhuis et al., 2014). Whether the rates of IFT change in aged neurons and/or how increased cilia length affects cilia signaling in neurons requires further investigation.

We also examined the cilia in the brains of the aged rats for the presence of SSTR3. We did not find statistically significant age effects in the numbers of SSTR3+ cilia in the hippocampi or neocortices of either aged F344 × BN or F344 rats. The function of the SSTR3 receptor in cilia is not well understood; however, studies of SSTR3 KO mice have found that these KO mice were more seizure-prone in the pentylenetetrazol kindling model (Qiu et al., 2008) suggesting that SSTR3 may play a role in regulating the excitability of cortical neurons. Our observation of persistent ciliary expression of SSTR3 in the CA1 subregion of the hippocampi of rats up to 34 months of age differs from a previous study of neuronal cilia in the hippocampi of rats up to 5 months of age that showed that SSTR3+ cilia were rarely detected in CA1 (Stanić et al., 2009). One possible explanation for this difference is that there may be strain differences in the regulation of SSTR3 expression in rodents; Stanić et al. (2009) examined Sprague-Dawley rat tissue whereas we examined F344 × BN rat tissue. It is noteworthy that in aged (20–24 month old) C57/BL6 mouse hippocampus, the distribution and length of SSTR3+ cilia in the DG of 20–24 month animals was similar to young (6–8 mos) and middle aged (14–18 mos) animals (Chakravarthy et al., 2012a), which was consistent with our findings in the DG. Alternatively it was recently reported that SSTR3 localization in mouse hippocampal neuronal cilia is dynamic, and that ligand exposure caused rapid decreases in SSTR3 ciliary localization (Green et al., 2016). Thus, depending on the extracellular environment, certain neuronal populations may fluctuate the levels of ciliary SSTR3 during the lifespan. Alternatively it is possible that aged neurons do not retain the ability to dynamically localize SSTR3 in response to ligand. While not examined in this study, it is possible that the localization of other GPCRs known to populate hippocampal neuronal cilia, such as MCHR1 (Sun et al., 2012), change with age. Changes in the types and levels of GPCRs localized to primary cilia could have functional consequences since MCHR1 and SSTR3 may form heterodimers within neuronal cilia (Green et al., 2012). It is interesting to speculate that aging could be associated with changes in the types and numbers of GPCRs in primary cilia, and that these changes produce unique signaling cascades within the ciliary microenvironment that are appropriate for the aging brain.

From the patterns of ACIII and SSTR3 immunostaining, we can also infer several other properties about cilia function that appear intact in the aged cortex. First, the presence of SSTR3 along the length of the cilium suggests that the function of the Bardet–Biedl Syndrome complex (BBSome), which contributes to IFT and trafficking of neuronal ciliary GPCRs, is intact (Nachury et al., 2007; Berbari et al., 2008b; Jin et al., 2010). The restricted localization of ciliary ACIII also suggests the transition zone is intact. The transition zone functions to separate the cilium from soma compartment and is a barrier permissive only to cilia-bound proteins (for review see: Garcia-Gonzazlo and Reiter, 2012), and mutations in transition zone-associated proteins are associated with reduced ACIII+ cilia frequencies (Stratigopoulos et al., 2014). The presence of pericentrin at the base of aged cilia suggests that the trafficking of proteins to the ciliary base is intact and that the cilia may continue to recruit IFT-related proteins to maintain cilia assembly (Jurczyk et al., 2004). The accumulation of pericentrin at the base of cilia also suggests that the pericentriolar material which forms around the basal body (for reviews see: Delaval and Doxsey, 2010; Mennella et al., 2014) is also intact. Future ultrastructural analyses, however, will need to be conducted to determine whether any underlying microtubule/structural deficits could be present in aged cilia.

In conclusion, our findings reveal that neurons in the aged cortex of the rat continue to elaborate primary cilia and that no major age-related changes in expression of known signaling molecules associated with neuronal cilia. Future questions that should be addressed are whether ACIII or SSTR3 signaling pathways are impaired with advanced age or whether there are any sex-related differences in these pathways as we did not examine female rats. Although aged neuronal cilia appear equipped with signaling machinery, do they maintain all of their signaling properties? Are there age-related cognitive deficits that correlate with changes in neuronal cilia? Future functional and mechanistic studies aimed at identifying additional receptors and signaling pathways in mammalian neuronal cilia and determining whether these signaling pathways are shared across different neuronal subtypes will lead to a better understanding of the function of neuronal cilia, information that could shed light on these important organelles and how they influence neuronal processes and functional outcomes during brain aging.

## Author Contributions

SMG contributed to the design, acquisition and interpretation of the data, and helped draft the manuscript. AKP contributed to the design, acquisition and interpretation of the data, and helped draft the manuscript. AS assisted in performing the experiments, analyzing the data and helping draft the manuscript. GFF assisted in performing the experiments, analyzing the data and helping draft the manuscript. GS provided critical reagents without which the study was not possible, and helped with interpretation and drafting of the manuscript. RJM assisted with data analysis and interpretation, and provided intellectual contribution during drafting of the manuscript. TCF helped with design and data interpretation, drafting the manuscript and provided funding support for the project. SS-R helped with drafting and editing the manuscript, data interpretation, and provided important intellectual contributions. AK helped with design, acquisition and interpretation of the data, and drafting the manuscript. MRS conceived the overall design of the study, and helped with data acquisition, analysis and interpretation, helped draft the manuscript, and provided funding support for the project.

## Conflict of Interest Statement

The authors declare that the research was conducted in the absence of any commercial or financial relationships that could be construed as a potential conflict of interest.

## Footnotes

^1^http://rsbweb.nih.gov/ij/
